# ﻿*Nicotianarupicola* sp. nov. and *Nicotianaknightiana* (sect.Paniculatae, Solanaceae), a new endemic and a new record for the flora of Chile

**DOI:** 10.3897/phytokeys.188.73370

**Published:** 2022-01-18

**Authors:** Ludovica Santilli, Fernanda Pérez, Claire De Schrevel, Philippe Dandois, Héctor Mondaca, Nicolás Lavandero

**Affiliations:** 1 Museo Nacional de Historia Natural, Área Botánica, Interior Parque Quinta Normal S/N, Casilla 787, Santiago, Chile Museo Nacional de Historia Natural, Área Botánica Santiago Chile; 2 Departamento de Ecología, Facultad de Ciencias Biológicas, Pontificia Universidad Católica de Chile, Santiago, Chile Pontificia Universidad Católica de Chile Santiago Chile; 3 Independent researcher, Copiapó, Chile Independent researcher Copiapó Chile; 4 Independent researcher, Santiago, Chile Independent researcher Santiago Chile

**Keywords:** Coquimbo, endemism, exotic species, Nicotianoidae, systematics, taxonomy

## Abstract

*Nicotianaknightiana* is recorded for the first time for the flora of Chile. A new species of *Nicotiana*, endemic to the coast of the Coquimbo region is described and illustrated. Molecular analysis placed the new species within the N.sect.Paniculatae, as sister to *N.cordifolia*, an endemic to Juan Fernandez islands. The new species can be considered critically endangered (CR) according to the IUCN categories due to its restricted and fragmented distribution, small population number, and the threat that urbanization and mining activities represent for the conservation of the biodiversity of the area.

## ﻿Introduction

*Nicotiana* L. is one of the largest genera in the Solanaceae with 75 recognised species (Clarkson et al. 2017) including the important crop plant *Nicotianatabacum* L. *Nicotiana* is naturally distributed in America and Australia, with one species from south-west Africa (Hunziker 2001). Evidence of homoploidy and polyploidy showed that hybridization has been a major driver of speciation in the genus (Clarkson et al. 2010).

Since Reiche’s (1903) treatment, no modern revision of *Nicotiana* in Chile has been published. According to the last catalogue of the flora of Chile (Rodriguez et al. 2018), the country is home to 12 native accepted species of *Nicotiana* distributed from Arica y Parinacota region to Magallanes region and Juan Fernandez Archipelago, and two introduced species, *Nicotianatabacum*, in Juan Fernandez and Easter Island, and *Nicotianaglauca* Graham in North and Central Chile. In his monograph of the genus, Goodspeed (1954) provided a taxonomic classification in which he recognised three subgenera further divided into 14 sections. Most of his sections have been confirmed as monophyletic by molecular analyses (Aoki and Ito 2000; Chase et al. 2003; Clarkson et al. 2004; Mehmood et al. 2020). Two Chilean species are included in Nicotianasect.Paniculatae Goodsp.: the endemics *Nicotianacordifolia* Phil. found in Juan Fernandez Archipelago (Philippi 1856) and *Nicotianasolanifolia* Walp., that grows between Tarapacá and Coquimbo regions (Walpers 1844) and has been traditionally used in the economic and cultural life of the Atacama coastal communities (Ballester et al. 2016). According to Goodspeed (1954), N.sect.Paniculatae share cordate-ovate leaves, evenly spaced along the stem, a long and narrow cylindrical thyrse with thickened central axis, a calyx with regular, broadly triangular teeth, a short and distinct tube proper with a several times longer throat, a greenish to yellowish corolla, stamens of almost equal length, filaments curving immediately above their insertion (except *N.cordifolia* and *N.raimondii* J. F. Macbr.), and anthers bending towards the stigma.

The aim of the present work is to record *Nicotianaknightiana* Goodsp. for the first time for the flora of Chile and describe a new species of *Nicotiana* endemic to Chile, determining its phylogenetic position and conservation status.

## ﻿Methods

### ﻿Herbarium and fieldwork

**Figure 1.**
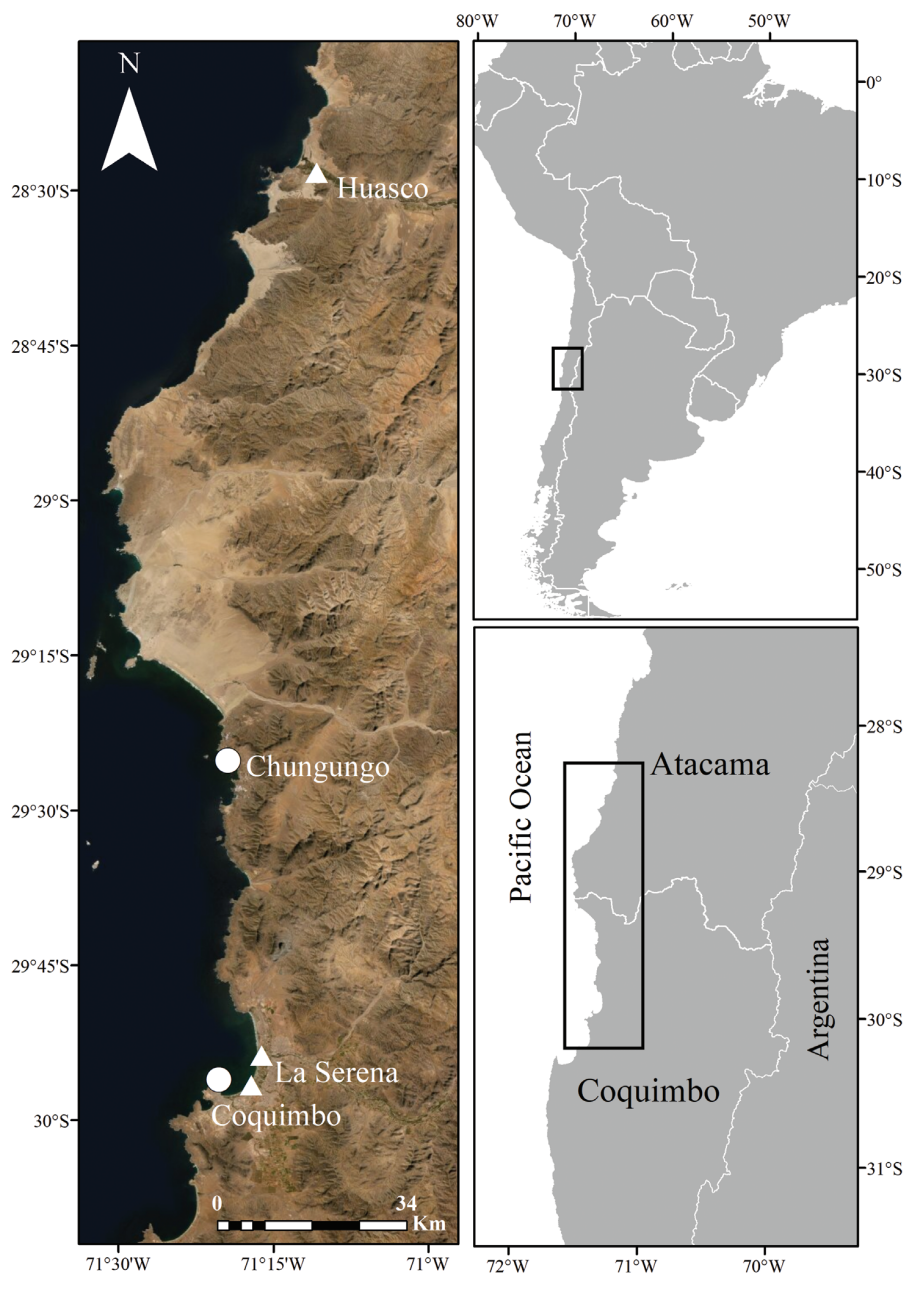
Distribution map of known locations of *Nicotianaknightiana* (triangles) and *Nicotianarupicola* (circles) in Chile. Service Layer Credits: Esri, DigitalGlobe, GeoEye, Earthstar Geographics, CNES/Airbus DS, USDA, USGS, AeroGRID, IGN, and the GIS User Community.

Fieldwork was carried out during November 2020 in Fuerte Lambert and during March 2020 in the proximity of the rivermouth of Elqui river, within the city of La Serena, Coquimbo region (Fig. 1). Specimens were collected and deposited in SGO herbarium. Physical and digital specimens of *Nicotiana*, including types, were revised at SGO, EIF, CORD, L, E, SI, F, NYBG, UC and US to reach a confident identification and check for possible previous collections of the species found in the field (acronyms according to *Index herbariorum*; http://sweetgum.nybg.org/science/ih/). Additionally, the citizen science platform iNaturalist (www.inaturalist.org) was consulted to search for possible observations of any of the two species. Terminology of the descriptions followed Goodspeed (1954). Accordingly, the corolla will be differentiated in three parts: the narrowest basal part of the corolla tube (the tube proper), the part of the corolla between the portion where the corolla tube broadens and up to the limb (throat), and the limb. The corolla tube is defined as the tubular portion of the corolla, from the receptacle up to the limb.

### ﻿Taxon sampling for phylogenetic analysis

DNA sequences for cpDNA intergenic spacers *trnF-trnL*, *trnS-trnG*, and genes *ndhF* and *matK* were obtained from GenBank (www.ncbi.nlm.nih.gov/Genbank) for all species of *Nicotiana* used in Clarkson et al. (2004). Sequences for the new species were generated in the present study. As outgroups we used *Symonanthusaromaticus* (C.A.Gardner) Haegi, and *Mandragoraofficinarum* L.

### ﻿DNA extraction, amplification, sequencing, and phylogenetic analyses

Total genomic DNA was extracted from silica-dried material collected in the field using the Qiagen DNeasy Plant Mini Kit (QIAGEN, Santiago, Chile), following the manufacturer’s instructions. Genomic DNA was used to amplify by PCR the following chloroplast regions: *trnL-trnF* using the primers c and f (Taberlet et al. 1991), *trnS-trnG* using the primers *trnS* (GCU) and *3’trnG* (UUC) (Hamilton 1999; Shaw et al. 2005), *ndhF* using the primers 972F and 2110R (Olmstead and Sweere 1994), *matK* using the primers pairs matK-TR and matK_4F, trnK-710F and matK-1848R, and matK_1F and matK_4R (Johnson and Soltis 1995; Aoki and Ito 2000; Bremer et al. 2002). We amplified all regions in 25 μl PCR reactions using the following thermocycling conditions: initial denaturation of 95 °C for 5 min; 35 cycles at 95 °C for 1 min, a specific annealing temperature for 1 min at 50 °C (60 °C for *trnS-trnG*), 72 °C for 1 min; and a final elongation period of 72 °C for 15 min. Sanger sequencing was performed using the same primers used during amplification in the case of regions *matK* and *trnL-trnF*, the same primers used during amplification plus the primers *ndhF*_1318 and *ndhF*_1603R (Olmstead and Sweere 1994) in the case of region *ndhF*, and the primers *trnS* (GCU) and *trnG* (UUC) (Hamilton 1999) for the *trnS-trnG* region. Sequencing was performed in the Plataforma de Secuenciación y Tecnologías Ómicas, Pontificia Universidad Católica de Chile, using the ABI PRISM 3500 xl Genetic Analyzer (Applied Biosystems). GenBank accession numbers for all DNA sequences are given in Suppl. material 1.

The assembled sequences were aligned using the MAFFT v7.450 algorithm (Katoh et al. 2002; Katoh and Standley 2013) in Geneious Prime 2021.1.1 (https://www.geneious.com). Phylogenetic analyses were run for both Maximum-likelihood (ML, Felsenstein 1981) and Bayesian inference (BI, Huelsenbeck and Ronquist 2001), using RAxML-AVX3 version (Stamatakis 2014) included in RAxMLGUI v.2.0 (Silvestro and Michalak 2012; Edler et al. 2020) and MrBayes x64 v3.2.7 (Ronquist et al. 2012), respectively. The best-supported model of nucleotide sequence evolution for each partition was determined based on the Akaike Information Criterion (AIC) using MrModeltest v2 (Nylander 2004). For the BI analysis, four partitions were used corresponding to each region, in which evolutionary models for each one were: GTR+G for *ndhF*, *trnL-trnF* and *trnS-trnG*, and GTR+I+G for *matK*. Maximum likelihood analyses were run using the GTRGAMMA approximation. The analysis included 1000 ML slow bootstrap replicates with 500 runs. Bayesian analyses were conducted under the respective best fit models for each partition, with two independent runs for 4 million generations, sampling every 1000 generations. Time series plots and effective sample size (ESS) were analysed using TRACER v.1.7 (Rambaut et al. 2018) in order to check convergence for each run. The first 1 million generations were discarded as burn-in.

### ﻿Conservation assessment

The assessment of the conservation status of the new species was made using the International Union for Conservation of Nature criteria (IUCN 2017). The extent of occurrence (**EOO**) and area of occupancy (**AOO**) were calculated using GeoCat (Bachman et al. 2011).

## ﻿Results

**Figure 2.**
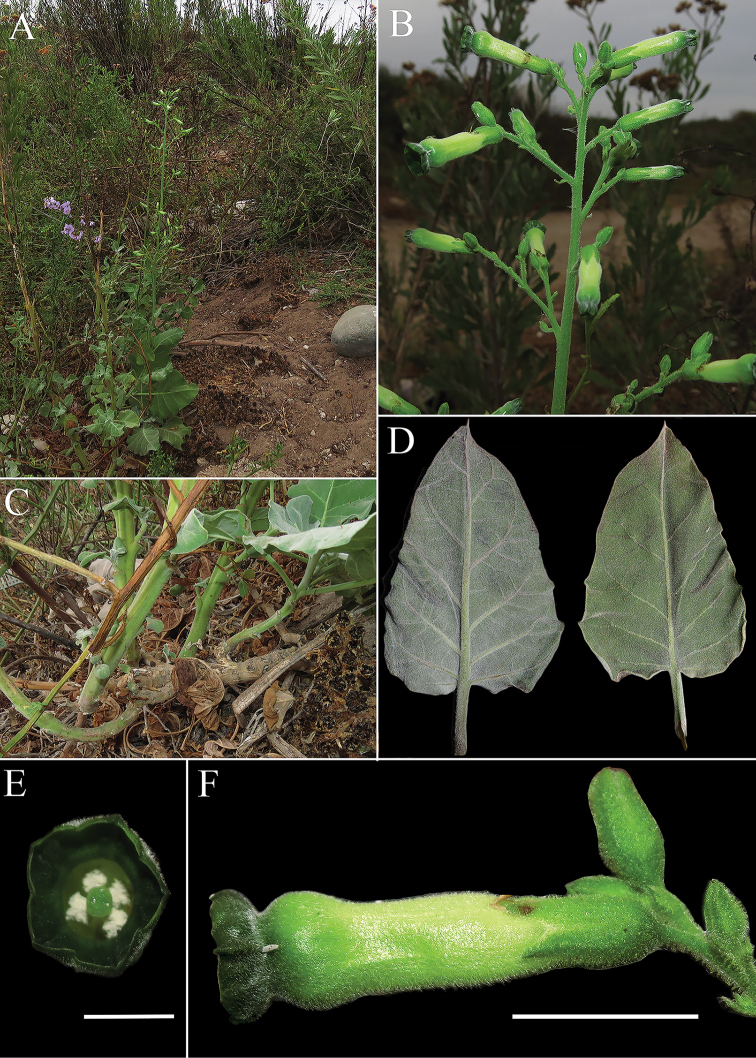
*Nicotianaknightiana* (*L. Santilli 210323)***A** habit **B** inflorescence **C** detail of lignified horizontal stem **D** adaxial and abaxial side of a leaf **E** frontal view of a flower showing limb area **F** lateral view of a flower. Scale bars: 1 cm.

**Figure 3.**
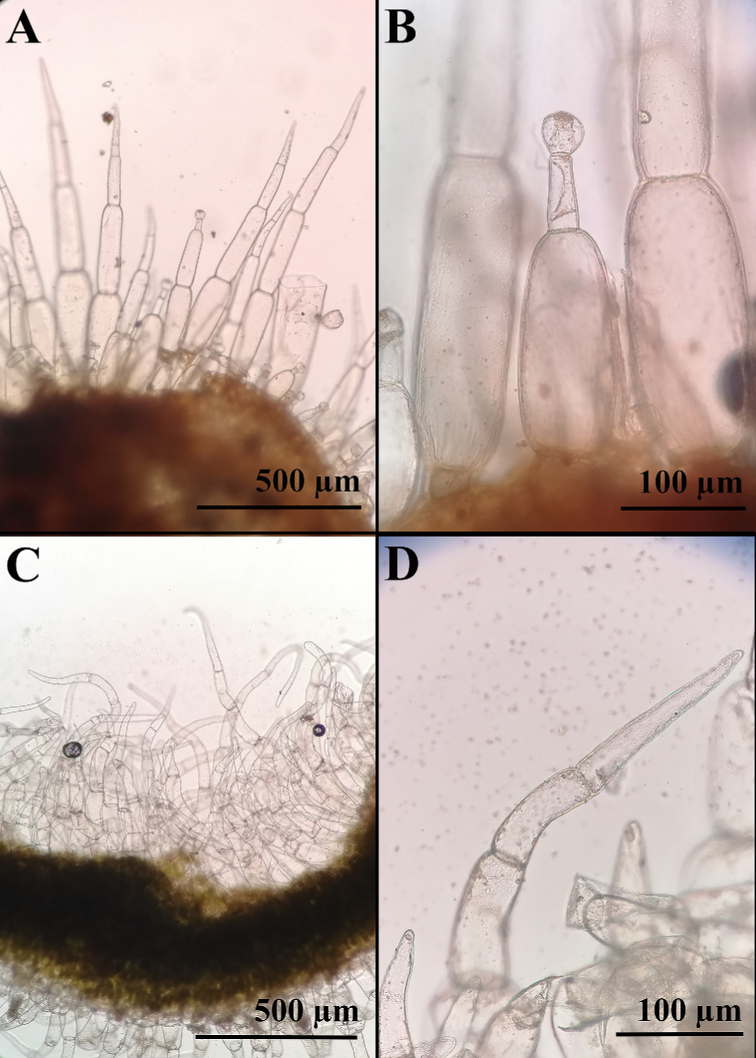
Indumentum of the leaves **A, B***Nicotianarupicola* with glandular hairs *(N. Lavandero 1011)***C, D***Nicotianaknightiana* with eglandular hairs (*L. Santilli 210323)*.

**Figure 4.**
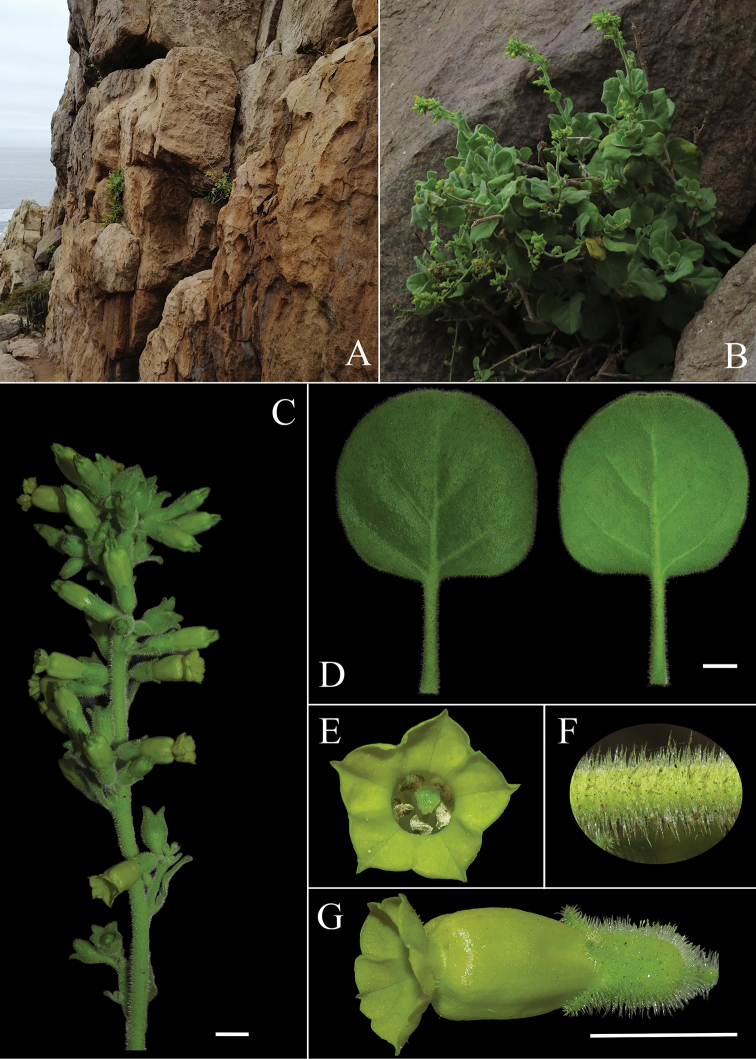
*Nicotianarupicola (N. Lavandero 1011)***A** habitat **B** habit **C** inflorescence **D** adaxial and abaxial side of a leaf **E** frontal view of a flower showing limb area **F** detail of indumentum **G** lateral view of a flower. Scale bars: 1 cm.

**Figure 5.**
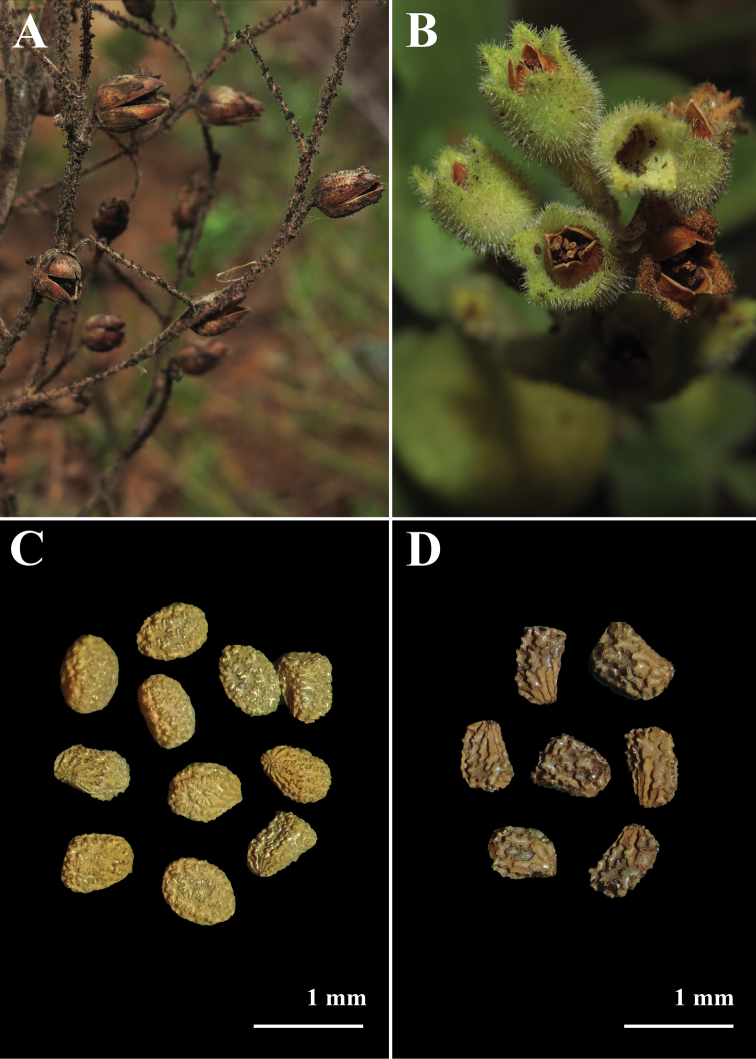
Fruits and seeds **A, C***Nicotianaknightiana* (*L. Santilli 210323)***B, D***Nicotianarupicola (N. Lavandero 1011)*.

We could not find any described species of *Nicotiana* for Chile that matched the morphology of the plants from Elqui river and Fuerte Lambert. A specimen matching the morphology of the species from Elqui river was found at SGO, collected in 2021 in the city of Huasco Bajo, Atacama, approx. 160 km north of La Serena. Plants from Elqui river are 2 m-long, somewhat ineffectively rooted, with branching short perennial shrubs, a pale yellow-green corolla, a dark green limb and eglandular indumentum (Fig. 2, Fig. 3C-D). This species was identified as *N.knightiana*, a member of Nicotianasect.Paniculatae known from coastal southern Peru. Two geo-referenced and misidentified observations of *N.knightiana* in Chile are available on iNaturalist, one from November 2018 in the proximity of our collection site (https://www.inaturalist.org/observations/19777218) and one from May 2021 at approx. 6 km southwards (https://www.inaturalist.org/observations/80390445) (Fig. 1). A specimen matching the morphology of the species from Fuerte Lambert was found at EIF, collected in 2006 in the whereabouts of Chungungo, Coquimbo, aprox 50 km north of Fuerte Lambert. Plants from Fuerte Lambert are perennial, rupicolous shrubs, with glandular indumentum, a characteristic congested inflorescence, and small, yellow flowers with an almost glabrous corolla (Fig. 3A-B, Fig. 4). Both species have capsules that produce a large number of seeds but the ones of *N.knightiana* are more rounded in shape compared to those of the new species (Fig. 5).

### ﻿Molecular phylogenetic analyses

**Figure 6.**
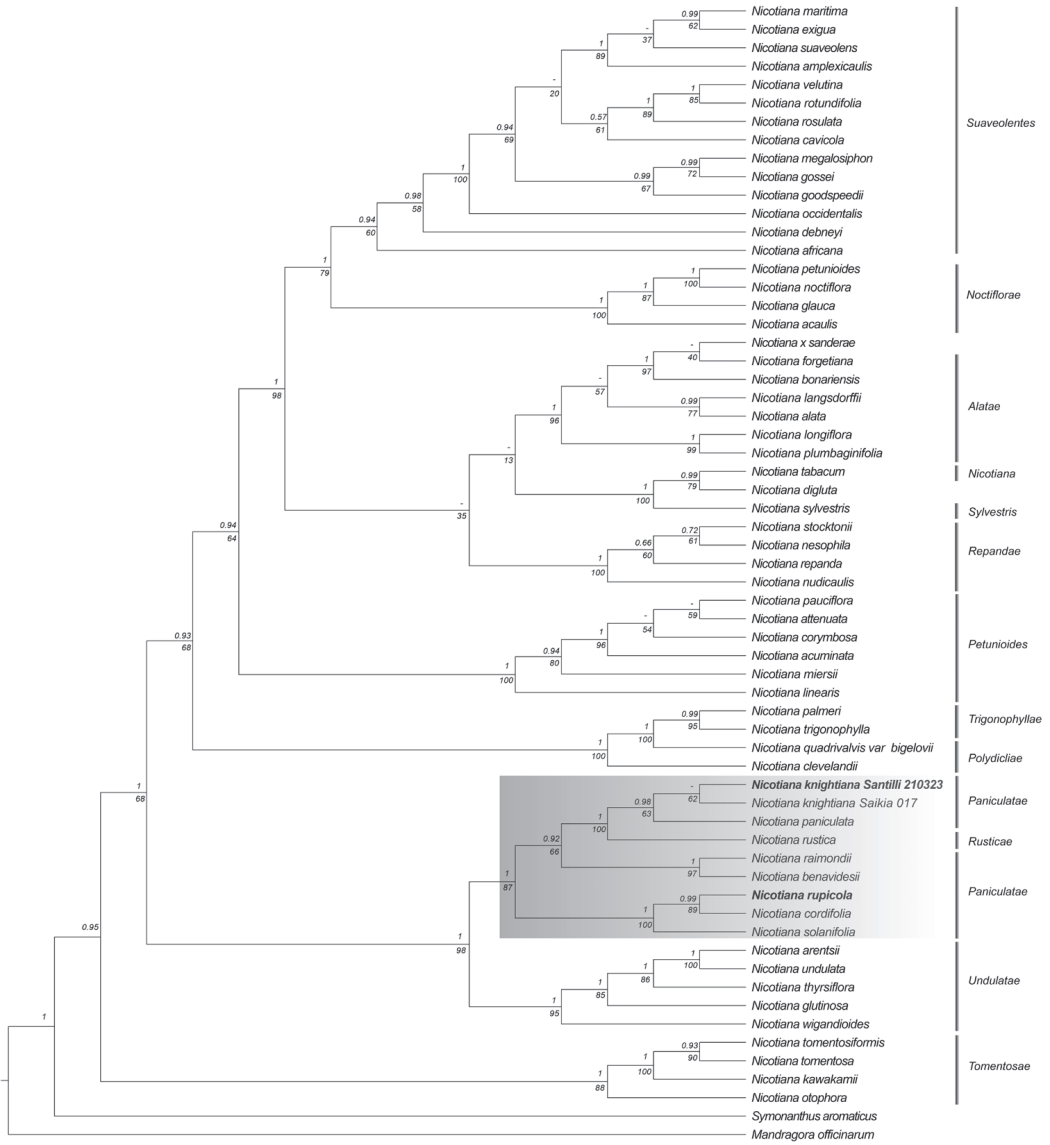
Phylogeny of *Nicotiana* resulting from Maximum Likelihood analysis of the plastid regions *matK*, *rps16*, *trnS-trnG* and *trnL-trnF*. Numbers above and below the branches represent the Posterior probabilities from the BI analysis and bootstrap values from the ML analysis, respectively. The species whose sequences were obtained in the present study are highlighted in bold, while sectionPaniculatae including *N.rustica* is highlighted in grey.

The concatenated DNA matrix contained 4427 nucleotide characters (1554 matK, 1074 *ndhF*, 932 *trnL-trnF* and 867 *trnS-trnG*), representing 60 ingroup and 2 outgroup accessions. Both BI and ML analyses yielded congruent topologies. The topology of the phylogenetic tree constructed in this study is congruent with the clades found by Clarkson et al. (2004) (Fig. 6). Overall, the support given by Bayesian posterior probabilities are higher than bootstrap values given by ML analyses. *Nicotiana* sections *Tomentosae* Goodsp. (PP = 1, BS = 88), *Undulatae* Goodsp. (PP = 1, BS = 95), *Paniculatae* (PP = 1, BS = 87), *Trigonophyllae* Goodsp. (PP = 1, BS = 100), Petunioides G.Don (PP = 1, BS = 100), *Alatae* Goodsp. (PP = 1, BS = 96), *Repandae* Goodsp. (PP = 1, BS = 100), *Noctiflorae* Goodsp. (PP = 1, BS = 100), and *Suaveolentes* Goodsp. (PP = 0.94, BS = 60) all form monophyletic groups with moderate to high supporting values.

SectionPaniculatae, including *N.rustica*, forms a well-supported clade (PP = 1.0, BS = 87). Relationships among the clades largely reflect Clarkson et al. (2004), including the position of the sectionPaniculatae as sister to sectionUndulatae and together as sister to the rest of the genus excluding sectionTomentosae. The new species falls within sectionPaniculatae as sister to *N.cordifolia* (PP = 0.99, BS = 89), and together they form a clade with *N.solanifolia* (PP = 1, BS = 100). This clade is sister to a clade including the rest of sectionPaniculatae (PP = 0.92, BS = 66). *Nicotianaknightiana* and *N.paniculata* are closely related species (PP = 0.98, BS = 63) and form a clade with *N.rustica* (PP = 1, BS = 100). The species collected in the mouth of Río Elqui, and identified as *N.knightiana*, falls as sister to *N.knightiana* sequenced in Clarkson et al. (2004) with moderate support (PP < 0.5, BS = 62). A base-by-base comparison of the sequences of *N.knightiana* from Clarkson et al. (2004) and *N.knightiana* from this study, show that they are identical, while they present differences at 6 nucleotide positions (3 from *trnS-trnG* and 3 from *matK* regions) with *N.paniculata*.

### ﻿Taxonomic treatment

#### ﻿ Nicotiana knightiana

Taxon classificationPlantaeSolanalesSolanaceae

Goodsp., Univ. Calif. Publ. Bot. 18: 139, pls. 11, 12b (1938).

66B5F5EC-8756-5CBD-90C0-F4CDA4F8204F

**Figure 7.**
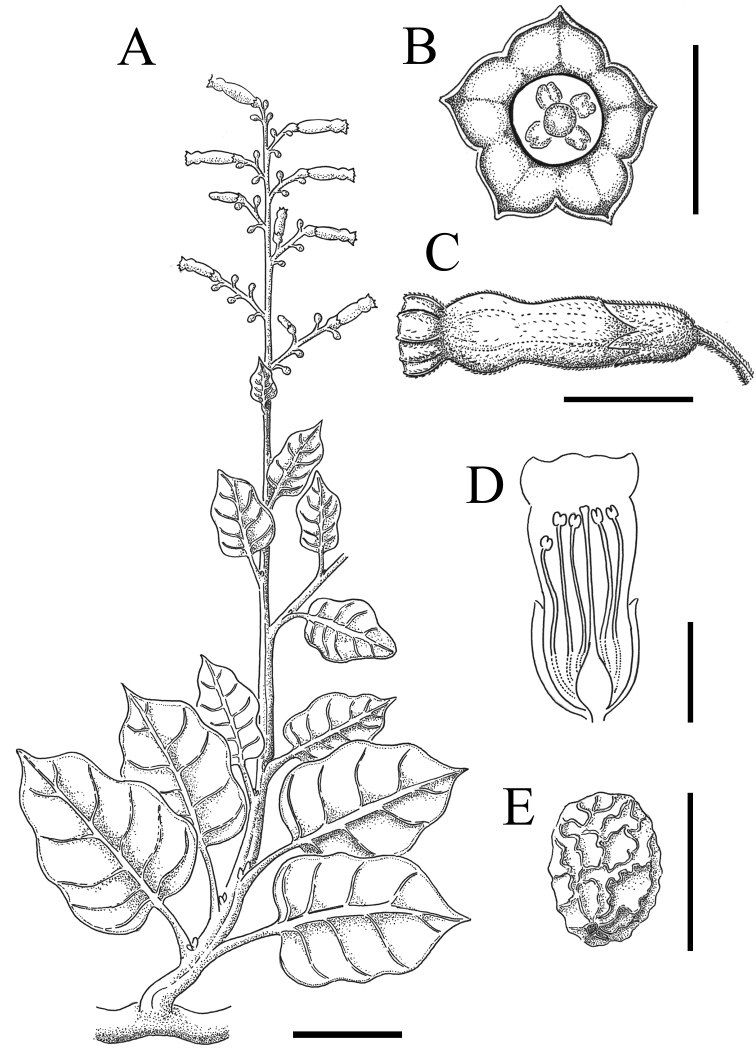
Illustration of *Nicotianaknightiana***A** entire branch **B** frontal view of a flower **C** lateral view of a flower **D** schematic drawing of a longitudinal section of a flower **E** seed. Scales: 5 cm (**A**); 0.5 cm (**B**); 1 cm (**C, D**); 0,5 mm (**E**). *Santilli 210323* (SGO).

Figures 2 , 3C–D , 5A–C , 7

##### Type.

Peru. Dept. Arequipa, Prov. Islay, near Mollendo, 40 m, 16 November 1935 (fl, fr), *Y. Mexia 04161* (holotype: UC [UC448735 photo!]; isotypes: MO, NA, NY).

##### Description.

Robust annual or short-lived shrub up to 3 m with many new stems at different stages of development arising from a lignified horizontal stem poorly anchored to the soil. ***Stems*** herbaceous, green, tomentose. ***Leaves*** ovate, undulate, base rounded to subcordate, apex obtuse to acute; bigger leaves 13 × 10 cm, indumentum similar to the stem but much denser on the abaxial side which confers a whitish colour, hairs simple, eglandular, pluricellular, brochidodromous venation, petioles a third or half as long as the leaves. ***Inflorescence*** a broad thyrse or lax panicle, 40 cm. Pedicels 0.5–1 cm in mature fruits, covered in glandular hairs. ***Calyx*** up to 7 mm, cylindric, tomentose, teethup to 2 mm, triangular. ***Corolla*** 20–23 mm excluding the limb (tubular part), tube proper 4 mm, throat 16 mm, pale yellow-green, covered in short, eglandular, hairs, limb bottom 3 mm wide, dark green, same indumentum as tube proper, notched into 5 lobes shorter than 1 mm. ***Stamens*** extending below the limb, 19 mm except one slightly shorter, filaments adnate to the tube proper, then free, pubescent in the proximal 6 mm, then glabrous and slightly curved, with stamens bending toward the stigma. ***Capsule*** 6–8 mm, ovoid. ***Seeds*** mainly subrotund, 0.5–0.7 mm, brown, surface reticulate. ***Embryo*** straight.

**Figure 8.**
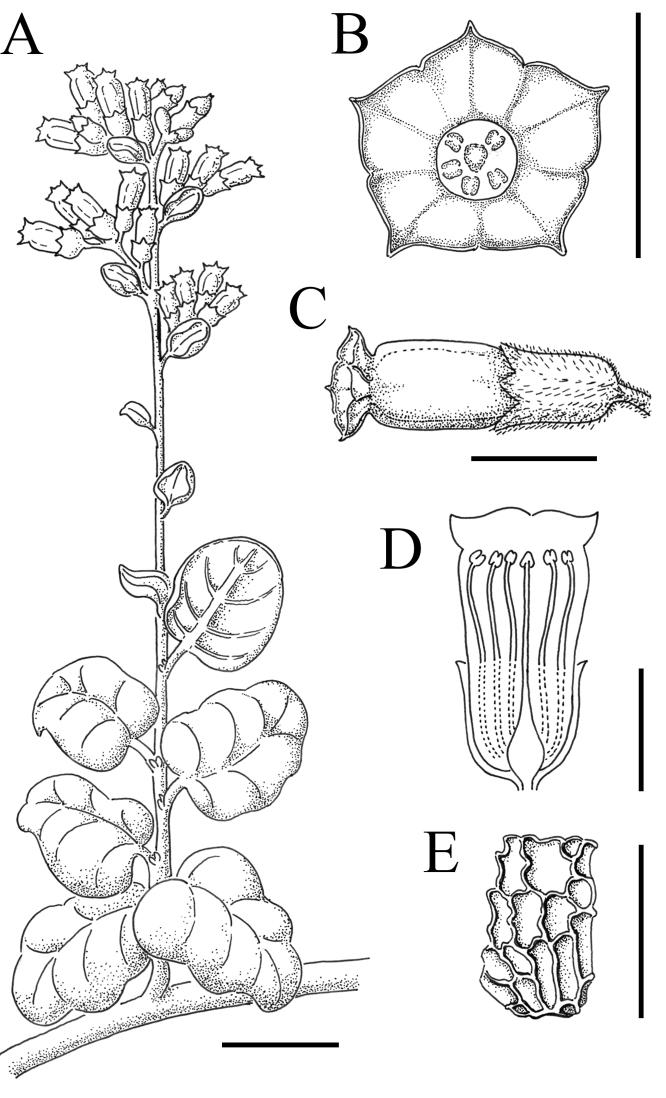
Illustration of *Nicotianarupicola***A** entire branch **B** frontal view of a flower **C** lateral view of a flower **D** schematic drawing of a longitudinal section of a flower **E** seed. Scales: 2 cm (**A**); 1 cm (**C, D**); 0.5 mm (**E**). *N. Lavandero* 1011 (SGO, EIF, CONC).

##### Distribution and habitat.

*Nicotianaknightiana* grows naturally in the coast of southern Perú in roadsides, pastures and rocky ravines bottoms. It was found in Chile, within the city of La Serena, Coquimbo region, in the proximity of the rivermouth of Rio Elqui, and in the proximity of Avenida Los Pescadores. It was also found growing in the city of Huasco Bajo, Atacama region (Fig. 1). It grows in a dense *Tessariaabsinthioides* (Hook. & Arn.) DC. scrub, associated with *Myoporumlaetum* G. Forst., *Phylanodiflora* (L.) Greene, *Schoenoplectuscalifornicus* (C.A. Mey.) Soják, *Solanumpinnatum* Cav., *Lyciumchilense* Miers ex Bertero, *Sarcocornianeei* (Lag.) M.A. Alonso & M.B. Crespo, *Distichlisspicata* (L.) Greene, *Thypaangustifolia* L., *Ambrosiachamissonis* (Less.) Greene, *Nicotianaglauca* and Stemodiadurantifolia (L.) Sw. var.chilensis (Benth.) C.C. Cowan. It thrives in sandy soils with water table very close to the surface.

##### Phenology.

*Nicotianaknightiana* is found flowering and fruiting between November and May.

##### Specimens examined.

Perú. Arequipa: Prov. Islay, Quebrada Canyon, 5–6 km north of Mollendo, 300 m, 29 September 1938 (fl, fr) *C.R. Worth & J.L. Morrison 15742* (US); 12 km southeast of Islay, 250–300 m, 28 September 1938 (fl, fr),*C.R. Worth & J.L. Morrison 15724* (US). Chile. Atacama: Huasco bajo, 28 m, 16 October 2021 (fl), *J.H. Macaya 1782* (SGO). Coquimbo: Prov. del Elqui, La Serena, ribera sur del Río Elqui a ca. 200 m de la desembocadura, 2 m, 23 March 2021 (fl, fr), *L. Santilli 210323* (SGO); La Serena, ribera sur del Río Elqui a ca. 200 m de la desembocadura, 1 m, 24 Nov 2018 (fl, fr), *A. Ryan* (iNaturalist); La Serena, avenida Los Pescadores con Canto del Agua, 3 m, 26 May 2021 (fl), *B.L. Bedard* (iNaturalist).

#### ﻿ Nicotiana rupicola

Taxon classificationPlantaeSolanalesSolanaceae

Santilli, De Schrevel, Lavandero & Dandois sp. nov.

B740DE53-5C20-5F26-A7B4-FF340BEF5317

urn:lsid:ipni.org:names:77248643-1

##### Type.

Chile. Región de Coquimbo: Prov. Elqui, Comuna de Coquimbo, Fuerte Lambert, 29°56'2.52"S, 71°20'16.46"W, 29 m, 12 November 2020 (fl, fr), *N. Lavandero 1011* (holotype: SGO!; isotypes: EIF!, CONC!).

##### Diagnosis.

*Nicotianarupicola* is most similar to *N.solanifolia*, from which it differs by its congested panicle (vs. lax panicle), its short and glabrous corolla up to 18 mm (vs. corolla of 35–50 mm, pubescent), non-retroflexed corolla limb (vs. retroflexed corolla limb), mature capsule included or slightly exserted from calyx, 6–10 mm (vs. more than half the length excluded from calyx at maturity, 12–18 mm).

##### Description.

Perennial shrub up to 2 m with many stems arising from a lignified horizontal stem. **Stems** lignified, light brown, glabrous. **Leaves** orbicular to ovate, flat to slightly ondulate, margins slightly revolute, base rounded to cordate, apex retuse to obtuse; bigger leaves 10 × 8.5 cm, reducing their size towards the apex, densely covered in two types of hairs in both sides: simple, straight, pluricellular, up to 1 mm long, and glandular (capitate), straight, pluricellular, 50–600 µm long, brochidodromous venation, petioles a quarter to half as long as the leaves, 0.5–2.5(–5.0) cm long. **Inflorescence** a compact panicle, up to 35 cm; pedicels up to 0.5 cm, same indumentum as the leaves. **Calyx** up to 10 mm, cylindric, same indumentum as the leaves, teeth up to 3.5 mm, triangular. **Corolla** 17–18 mm excluding the limb (tubular part), tube proper up 5.5–6 mm, throat up to 12 mm, yellow, glabrous, limb 4 mm wide, yellow, glabrous or with scattered hairs, notched into 5 lobes. **Stamens** extending below the limb, similar length; filaments adnate for the first 5 mm to the tube proper, then free, pubescent at the base, then glabrous and slightly curved, with the distal portion bending toward the stigma. **Capsule** 6–10 mm long, ovoid. **Seeds** mainly angular, laterally compressed, 0.4–0.6 mm long, dark brown, surface reticulate. **Embryo** unknown. **Chromosome number** unknown. (Fig. 3; Fig. 4B-D; Fig. 8)

##### Distribution and habitat.

*Nicotianarupicola* is endemic to Chile where it is currently known from two locations, Fuerte Lambert and Chungungo, both in the region of Coquimbo (Fig. 1). In Fuerte Lambert it grows among the rocks in a cliff near the ocean together with *Alstroemeriamagnifica* Herb., *Cistanthegrandiflora* (Lindl.) Schltdl, *Diplolepisboerhaviifolia* (Hook. & Arn.) Liede & Rapini, *Eulychniabreviflora* Phil., *Loasaelongata* Hook. & Arn., *Myrcianthescoquimbensis* (Barnéoud) Landrum & Grifo, *Nolanaacuminata* (Miers) Miers ex Dunal, *N.rupicola* Gaudich., *N.sedifolia* Poepp., *Ophryosporustriangularis* Meyen, *Plumbagocaerulea* Kunth, *Polyachyruspoeppigii* Kuntze ex Less., *Puyavenusta* Phil., Sicyosbaderoa Hook. & Arn. var.baderoa, *Solanumpinnatum* Cav., *Stachyspannosa* Phil., In Chungungo it grows on a rocky cliff facing the ocean, together with *Nolanacrassulifolia* Poepp. and *N.sedifolia* Poepp and surrounded by a scrub of *Balbisiapeduncularis* (Lindl.) D. Don, *Heliotropiumstenophyllum* Hook. & Arn., and *Oxalisvirgosa* Molina.

##### Phenology.

*Nicotianarupicola* was found flowering and fruiting in November.

##### Etymology.

The specific epithet derives from the Latin *rupes* (rock), and *cola* (dweller), alluding to rocky habitat of this species.

##### Additional specimens examined (paratypes).

Chile. Región de Coquimbo. Prov. Elqui, Comuna La Higuera, costa al Norte de Chungungo, 7 November 2006 (fl), *N. García 3085* (EIF).

##### Conservation status.

*Nicotianarupicola* can be considered as Critically Endangered (CR) under the IUCN categories and criteria B1ab(iii); D. The criterion B1 was selected because its extent of occurrence is < 100 km^2^ (8 km^2^). The criterion “a” was selected because the distribution is highly fragmented. The criterion “b(iii)” was selected because there is a projected decline in the area, extent and quality of habitat. This area is constantly threatened by the expansion of urbanization that is affecting central-north coastal Chile. One of the locations is currently found at less than 300 m from the residential area of Coquimbo and the habitat is being altered by numerous and increasing amounts of formal and informal paths and human activity (camping, garbage, etc.). Moreover, mining activities within the extent of occurrence, especially Minera Dominga, which pretends to settle between the two known localities, will more likely affect possible unknown populations and the quality of the habitat. The criterion D was selected because we observed less than 50 individuals around the two known localities.

### ﻿Key to the species of Nicotianasect.Paniculatae found in Chile

**Table:** 

1	Inflorescence a congested panicle, corolla tube glabrous except for sparse hairs on the limb	***N.rupicola***
–	Inflorescence a lax panicle, corolla tube entirely covered by trichomes	**2**
2	Corolla tube 3–5 cm, indumentum of corolla made of glandular trichomes	***N.solanifolia***
–	Corolla tube 2–2.3 cm, indumentum of corolla made of eglandular trichomes	**3**
3	Corolla tube 2 cm, limb dark green, continental Chile	***N.knightiana***
–	Corolla tube 2–3 cm, limb yellow or purple, Juan Fernandez Islands	**4**
4	Corolla tube purple, Alejandro Selkirk Island	**N.cordifoliasubsp.cordifolia**
–	Corolla tube yellow, Santa Clara Island	**N.cordifoliasubsp.sanctaclarae**

## ﻿Discussion

The characters that proved to be useful to differentiate species of Nicotianasect.Paniculatae are the type of inflorescence, the types of trichomes and distribution of the indumentum, as well as the size and colour of flowers. *N.knightiana* resembles most *N.paniculata* from which it differs on account of its shorter flowers with dark green limbs (vs. yellow) (Fig. 2E-F) . The large undulate leaves, the narrow panicles and the long throat of the flowers of *N.knightiana* also resemble *N.solanifolia* from which it can be distinguished for its smaller flowers (2–2.3 cm vs 3–5 cm respectively), eglandular indumentum (vs. glandulous) and dark green limb (vs. yellow) (Figs 2E, F, 3C, D). *Nicotianarupicola* is morphologically similar to *N.solanifolia* from which it can be distinguished by its congested panicle (vs.lax), glabrous corolla tube (vs. hairy), its smaller flowers and no-retroflexed corolla (Fig. 4) .

It is worth mentioning that *N.solanifolia* has been erroneously reported for the Coquimbo region based on a collection held at K (*Cuming 860*), which is the type of *Nicotianabreviloba* Jeffr., considered by Goodspeed (1954) as a synonym of *N.solanifolia*. Johnston (1936) considered that Cuming’s collections with collection number between 853 and 911 were obtained north of Huasco, Atacama region. This is consistent with the collection localities of the specimens of *N.solanifolia* revised in the present work which show that the distribution of *N.solanifolia* and of *N.rupicola* do not overlap. The southernmost collection of *N.solanifolia* is found at approx. 100 km north of the northernmost collection locality of *N.rupicola*.

Molecular analyses showed that plants from Elqui river are likely correctly identified as *N.knightiana* and that our initial conjectures about the phylogenetic position of *N.rupicola* as part of the sect.Paniculatae were confirmed (Fig. 6) . Our topology retrieves Clarkson’s et al. (2004) results of two separate clades within the sect.Paniculatae that reflect geography, one including species from Peru and one including the two endemics *N.solanifolia* from the North of Chile and *N.cordifolia* from Juan Fernández Archipelago (Fig. 6). Species of Chilean sect.Paniculatae share long tubular flowers and long-petiolate leaves. Surprisingly, *N.rupicola* results in being more closely related to the Juan Fernández species *N.cordifolia* than to the continental species *Nicotianasolanifolia*, despite their morphological affinity.

An important question regards whether *N.knightiana* has to be considered native or introduced to Chile. Either the species was never noticed or collected during the last two centuries of botanical expeditions, and presents naturally disjunct populations, being almost 1500 km apart from the closest population found in Peru, or it was recently introduced in Chile by anthropogenic means. The earliest evidence of its presence dates back to 2018 (iNaturalist) and it seems to have been established and possibly expanded to the surrounding area in sites with similar ecological conditions to the river mouth of Elqui river, such as Huasco Bajo. The production of abundant and small seeds, together with the ability of some species to grow in a broad range of open and disturbed habitats, is considered as a common adaptation that ensures high probability of dispersal and establishment. Such is the case for *Nicotianapaniculata*, *N.glauca*, and *N.plumbaginifolia* Viv. that are considered invasive species (Gallo et al. 2008; Gairola et al. 2016; Rodríguez-Caballero et al. 2020; Alharthi et al. 2021)

*Nicotianarupicola* presents a restricted distribution limited to a small portion of the coastal area of northern Chile, where it grows on two locations on easily accessible coastal rocky cliffs at less than 300 m from urbanization. The population from Fuerte Lambert is situated in an area where urban expansion has caused major damage to the vegetation. The coastal area between Tongoy and Coquimbo is catalogued as a site of interest for the conservation of woody and succulent species due to its high diversity and rates of endemism (Squeo 2000). The area is home to various threatened species such as: *Caricachilensis* (Planch. ex A. DC.) Solms, *Myrcianthescoquimbensis* (Barnéoud) Landrum & Grifo, *Porlieriachilensis* I.M. Johnst. Controversially, it is an area particularly affected by intense expanding urbanization that seriously threatens the conservation of the local biodiversity. Additionally, a new mining facility and a discharge port (Minera Dominga) will be constructed within its extent of occurrence, close to its northernmost known locality. It is likely that several more individuals of *N.rupicola* are present in the area, since the abiotic and biotic conditions are similar to the currently known localities. None of these individuals were considered during the environmental impact assessment of the project. It is of great importance for the conservation of the species to search for more individuals and localities, and to raise the attention to much needed conservation measures for the species and the unique ecosystem where it is found.

### ﻿Additional specimens examined

*Nicotianasolanifolia*. **Chile. Antofagasta**: [Antofagasta Province]. Lomas de Taltal, near road from Taltal to the panamericana, 430 m, 25 October 2002 (fl), *M. Ackermann 500* (SGO); bei Hueso Parado, nahe von Taltal, 400 m, 9 July 1972 (fl, fr), *O. Zöllner 5942* (L); Quebrada de Taltal, 410 m, 17 September 1992 (fl, fr), *S. Teillier*, *P. Rundel & P. García 2850* (F); Hueso Parado, *s.d*., *s.c.* (SGO); Ravine ca. 16 km north from Paposo, 207 m, 21 November 2008 (fl), *R. Baines*, *M. Gardner*, *P. Hechenleitner*, *C. Morter & D. Rae 38.* (E); Mirador above the Thermoelectric plant below Quebrada Paposo, 680 m, 1 December 2004 (fl, fr), :*M. Dillon & M. Finger 8670* (SGO); Paposo, Peralito, 15 November 1959 (fl), *A. Torres s.n.* (SGO); Paposo, borde quebrada, 24 October 2009, *A. Moreira & F. Luebert 1205* (SGO); Camino Paposo-Caleta Blanco Encalada, Queb. Miguel Diaz, 160 m, 15 November 1996, *R. Rodriguez 3131* (SGO); El Rincón, al N de Paposo, 17 September 1941 (fl, fr), *C. Muñoz & G. Johnson 2877* (SGO); Quebrada el Despoblado, 25–26 August 1992 (fl), *J.C. Torres s.n.* (SGO); Taltal-Paposo, September 1909 (fl, fr), *K. Reiche s.n.* (SGO); Paposo, entrada a la Qda. Los Peralitos, 30 September 2005 (fl, fr), *M. Muñoz 4607* (SGO); 10 km al sur de Caleta Blanco Encalado, 200–800 m, 11 December 1940 (fl), *W. Biese 3209* (SGO). Taltal, Quebrada de Taltal, 410 m, 17 September 1992 (fl, fr), *S. Teiller*, *P. Rundel*, *P. García 2837* (SGO); 6 Km east of Taltal, 300–600 m, 14 October 1938 (fl, fr), *C.R. Worth & J.L. Morrison 16122* (US); Cerro Perales, ca. 5 km E of Taltal, 550–960 m, 27 September 1988 (fl, fr), *M.O. Dillon*, *D. Dillon & V. Pobleto 5536* (F); Quebrada Rinconada, ca. 5 Km N of Caleta Paposo, 250 m, 25 October 1988 (fl), *M.O. Dillon*, *D. Dillon & B. Tay 5741* (F);

**Atacama**: [Chañaral Province]. Parque Nacional Pan de Azúcar, Quebrada de Coquimbo, 10 November 1987, *Paez s.n.* (SGO); Chañaral, 24 October 1985 (fl), *G. Nieuwenhuizen 132*–*27* (SGO); Camino Chañaral a Flamenco, 3.5 km al interior camino izquierdo desde Portofino, quebrada y cono de deyección, 14 October 1992 (fl, fr), *M. Muñoz 3095* (SGO). [Copiapó Province]. Caldera, Quebrada León, 20 m, October 1924 (fl), *E. Werdermann*. *437* (E, SI, F); Quebrada de los leones, Caldera, 1888 (fl), *W. Geisse s.n.* (Type of *Nicotianacardiophylla* Phil.) (SGO); Caldera, September 1900, *K. Reiche s.n.* (SGO). [Huasco Province]. Camino Carrizal Bajo – Huasco, 30 m, 13 October 1991 (fl, fr), *S. Teillier*, *L. Villaroel & R. Torres 2579* (SGO); sector Aguada Tongoy, road to Los Bronces near Corral El Sauce – road junction, 276 m, 6 December 2004 (fl, fr), *P. Baxter*, *M. Gardner*, *P Hechenleitner*, *P.I. Thomas & C. Zamorano 1877* (E, SGO); Carrizal Bajo, September 1885 (fr), *F. Philippi s.n.* (SGO); Camino Carrizal Bajo, km 50, 2 November 1991 (fl, fr), *M. Muñoz*, *S. Teillier & I. Meza 2944* (SGO); Camino de vuelta Carrizal a Canto de Agua en Qda. Exposición sur, 23 September 1977 (fl, fr), *M. Muñoz 1119* (SGO); Carrizal, September 1885, *F. Philippi s.n.* (SGO).

*Nicotianacordifolia*. **Chile. Valparaíso**: [Valparaíso Province]. Archipiélago de Juan Fernandez, Isla Santa Clara, Bahía Matriz, 12 December 1998 (fl, fr), *P. Danton s.n*. (SGO); Isla Masafuera, s.d. (fl), *R. A. Philippi 730* (F), Isla Masafuera, October 1854 (fl, fr), *P. Germain s.n*. (SGO); Isla Masatierra, San Juan Bautista, Conaf Garden, 56 m, 13 December 2003 (fl, fr), *M. Gardner*, *P. Hechenleitner & M. Tobar* (E)

## Supplementary Material

XML Treatment for Nicotiana knightiana

XML Treatment for Nicotiana rupicola

## Supplementary materials

Supplementary material 1 GenBank accession numbers Data type Xlsx file. Explanation note GenBank accession numbers for the trnL-trnF, trnS-trn-G, matK and ndhF sequences used in this study. GenBank accessions in bold are new to this study. https://binary.pensoft.net/file/635299This dataset is made available under the Open Database License (http://opendatacommons.org/licenses/odbl/1.0/). The Open Database License (ODbL) is a license agreement intended to allow users to freely share, modify, and use this Dataset while maintaining this same freedom for others, provided that the original source and author(s) are credited.Ludovica Santilli, Fernanda Pérez, Claire De Schrevel, Philippe Dandois, Hector Mondaca, Nicolás Lavandero
